# Genome-wide association mapping of rust resistance in *Aegilops longissima*

**DOI:** 10.3389/fpls.2023.1196486

**Published:** 2023-07-27

**Authors:** Rae Page, Shuyi Huang, Moshe Ronen, Hanan Sela, Amir Sharon, Sandesh Shrestha, Jesse Poland, Brian J. Steffenson

**Affiliations:** ^1^ Department of Plant Pathology, University of Minnesota, Saint Paul, MN, United States; ^2^ Institute for Cereal Crops Research, Tel Aviv University, Tel Aviv, Israel; ^3^ School of Plant Sciences and Food Security, Tel Aviv University, Tel Aviv, Israel; ^4^ Department of Plant Pathology, Kansas State University, Manhattan, KS, United States; ^5^ Plant Science Program, Biological and Environmental Science and Engineering Division, King Abdullah University of Science and Technology (KAUST), Thuwal, Saudi Arabia; ^6^ KAUST Center for Desert Agriculture, King Abdullah University of Science and Technology, Thuwal, Saudi Arabia

**Keywords:** *Aegilops longissima*, genome-wide association mapping, wheat rust, plant disease resistance, genetic diversity, wild wheat relatives, germplasm collections

## Abstract

The rust diseases, including leaf rust caused by *Puccinia triticina* (*Pt*), stem rust caused by *P. graminis* f. sp. *tritici* (*Pgt*), and stripe rust caused by *P. striiformis* f. sp. *tritici* (*Pst*), are major limiting factors in wheat production worldwide. Identification of novel sources of rust resistance genes is key to developing cultivars resistant to rapidly evolving pathogen populations. *Aegilops longissima* is a diploid wild grass native to the Levant and closely related to the modern bread wheat D subgenome. To explore resistance genes in the species, we evaluated a large panel of *Ae. longissima* for resistance to several races of *Pt*, *Pgt*, and *Pst*, and conducted a genome-wide association study (GWAS) to map rust resistance loci in the species. A panel of 404 *Ae. longissima* accessions, mostly collected from Israel, were screened for seedling-stage resistance to four races of *Pt*, four races of *Pgt*, and three races of *Pst*. Out of the 404 accessions screened, two were found that were resistant to all 11 races of the three rust pathogens screened. The percentage of all accessions screened that were resistant to a given rust pathogen race ranged from 18.5% to 99.7%. Genotyping-by-sequencing (GBS) was performed on 381 accessions of the *Ae. longissima* panel, wherein 125,343 single nucleotide polymorphisms (SNPs) were obtained after alignment to the *Ae. longissima* reference genome assembly and quality control filtering. Genetic diversity analysis revealed the presence of two distinct subpopulations, which followed a geographic pattern of a northern and a southern subpopulation. Association mapping was performed in the genotyped portion of the collection (n = 381) and in each subpopulation (n = 204 and 174) independently via a single-locus mixed-linear model, and two multi-locus models, FarmCPU, and BLINK. A large number (195) of markers were significantly associated with resistance to at least one of 10 rust pathogen races evaluated, nine of which are key candidate markers for further investigation due to their detection via multiple models and/or their association with resistance to more than one pathogen race. The novel resistance loci identified will provide additional diversity available for use in wheat breeding.

## 1 Introduction

Numerous biotic stressors continually threaten the world’s supply of wheat. Most notorious are the rust diseases which continue to be an important limiting factor in wheat production in many regions of the world (Figueroa et al., 2018). These fungal diseases include leaf rust caused by *Puccinia triticina* (*Pt*), stem rust caused by *P. graminis* f. sp. *tritici* (*Pgt*), and stripe rust caused by *P. striiformis* f. sp. *tritici* (*Pst*).

Leaf rust is the most common rust disease, affecting a larger acreage of wheat per year worldwide than either stem or stripe rust (Bolton et al., 2008). Losses caused by leaf rust over large areas are generally light to moderate (1-20%); however, early infections on susceptible cultivars can be devastating in localized areas (USDA-ARS, 2019). The leaf rust pathogen displays considerable diversity for virulence on wheat. Over 70 races of *Pt* have been identified from nationwide collections in the United States by the USDA-ARS. In a worldwide collection of *Pt* isolates, 424 multilocus genotypes and 497 virulence phenotypes were found (Kolmer et al., 2019).

Stem rust has a long history of causing devastating epidemics worldwide (Chaves et al., 2008). In the hard red spring wheat-growing regions of North America, rust resistance has been stable for over 50 years; however, notable “boom and bust” cycles of resistance deployment and subsequent emergence of virulent races of the pathogen occurred multiple times prior to the current long-standing resistance (Ellis et al., 2014). Stem rust now threatens the world’s wheat germplasm due to the emergence of the widely virulent *Pgt* Ug99 lineage of races (e.g. TTKSK), first discovered in Uganda in 1998 (Pretorius et al., 2000). This race group was virulent on over 80% of the world’s wheat cultivars and breeding materials (Singh et al., 2011). Additional races unrelated to Ug99 have recently emerged in various regions, such as Ethiopia, Germany, and Italy (Olivera et al., 2015; Bhattacharya, 2017; Olivera Firpo et al., 2017), overcoming newly identified sources of resistance, and underscoring the difficulty of breeding for durable resistance to stem rust.

The third major rust disease is stripe rust, a quickly growing problem in many wheat production regions. In the United States, stripe rust is generally more restricted in its distribution; however, it has become increasingly more important over the past 20 years. It has now been reported in over 25 states (Wan et al., 2016) and in 2010 caused a severe epidemic in the Pacific Northwest. Worldwide, stripe rust has been responsible for damaging epidemics in nearly all wheat producing areas (Chaves et al., 2008). Over 70 stripe rust resistance genes have been described in wheat; however, only two (*Yr5* and *Yr15*) are effective against all races currently identified in the United States (Li et al., 2011).

Breeding for resistance to rust pathogens is key to delivering the most cost-effective and sustainable disease control method. Due to the highly fluid and adaptive nature of rust pathogen populations, many rust resistance genes within the primary gene pool of wheat have been overcome; thus, there is a need to expand the search for resistance to the secondary and tertiary gene pools. The secondary gene pool of wheat includes wild relatives that may be hybridized with cultivated wheat, but proper pairing and recombination of chromosomes must be artificially induced (Harlan and de Wet, 1971). Thus, there is considerable genetic diversity available for wheat improvement in the secondary and tertiary gene pools, but careful screening and proper utilization of the most closely related species is critical.

The allohexaploid bread wheat genome arose via two ancient hybridization events and contains three subgenomes (A, B, and D) (Marcussen et al., 2014). Initially, it was hypothesized that the wheat B subgenome originated from an ancestor of one of the five diploid *Aegilops* species of the section Sitopsis (carrying S or S* genomes), including *Ae. bicornis* (Spach goatgrass), *Ae. longissima* (elongated goatgrass), *Ae. searsii* (Sears’ goatgrass), *Ae. sharonensis* (Sharon goatgrass), and *Ae. speltoides* (truncate goatgrass). Recently, through genome sequencing and assembly of species in section Sitopsis, the origin of the B genome was hypothesized to be an extinct diploid species that diverged from an ancestral progenitor of the B lineage to which the extant *Ae. speltoides* and *Ambylopyrum muticum* (synonym: *Ae. mutica*) belong (Avni et al., 2022; Li et al., 2022). The remaining four Sitopsis species were shown to be members of the common wheat D subgenome lineage. The five Sitopsis species have been valuable sources of disease resistance genes. Numerous instances of successful transfers of disease resistance genes from the Sitopsis species into tetraploid and/or hexaploid wheat have been reported, including leaf rust resistance genes *Lr28*, *Lr35*, *Lr36*, *Lr47*, *Lr51* (all from *Ae. speltoides*), and *Lr56* (*Ae. sharonensis*); stem rust resistance genes *Sr32*, *Sr39*, *Sr47* (all from *Ae. speltoides*), *Sr62* (*Ae. sharonensis*), and *Sr51* (*Ae. searsii*); the stripe rust resistance gene *Yr38* (*Ae. sharonensis*); and powdery mildew resistance genes *Pm12*, *Pm32* (both from *Ae. speltoides*), and *Pm13* (*Ae. longissima*) (Schneider et al., 2008; Olivera and Steffenson, 2009; Liu et al., 2011; Klindworth et al., 2012; Yu et al., 2022).

*Aegilops longissima* (2*n* = 2*x* = 14, S^l^S^l^) is native to the Levant being distributed in the eastern Mediterranean coastal areas of Israel and southern Lebanon, with a limited distribution in the northern Sinai coast of Egypt extending to the coastal region around Alexandria. Further inland populations also exist in the Negev desert of southern Israel, and in limited distributions in southern Syria and northwestern Jordan (van Slageren, 1994). Its habitat preference is on sandy loam and “Hamra” soils (red sandy loam) near the seashore or loess soil and sandstone in the Negev desert, and terra rossa soils in Jordan (Sela et al., 2018). Accessions of *Ae. longissima* have been reported to carry resistance to leaf rust (Anikster et al., 2005), stem rust (Anikster et al., 2005; Scott et al., 2014), stripe rust (Anikster et al., 2005), powdery mildew (Li et al., 2020), and eyespot (Sheng et al., 2014). In spite of the demonstrated diversity of disease resistance in *Ae. longissima*, it nevertheless remains underutilized as a resource for discovery of novel rust resistance genes. Recently an extensive collection of *Ae. longissima* accessions was assembled (the *Ae. longissima* Diversity Collection; ALDIVCO) and evaluated for resistance to two races of *Pt* (BBBDB and THBJG), two races of *Pgt* (TTTTF and TTKSK), and one race of *Pst* (PSTv-37) (Huang et al., 2018). The results revealed that *Ae. longissima* was a rich source of resistance to all three rusts. Notably, 10 accessions were found to be resistant to all races of the three pathogens investigated in the study. The ALDIVCO is a promising resource for discovery of novel sources of resistance to all three rust pathogens. However, there remains a need to conduct additional rust evaluations in order to characterize the resistance spectrum of individual accessions and to elucidate the genetic basis of resistance.

Genome-wide association studies (GWAS) enable detection of marker-trait associations in genetically diverse populations by exploiting low linkage disequilibrium (LD) which occurs due to accumulated historic recombination events (Sorrells and Yu, 2009). This method is an efficient means for mapping multiple traits in a given population, even those comprised of wild germplasm like the ALDIVCO. Population structure, or the mixture of multiple subpopulations within a germplasm panel, must be taken into account when performing a GWAS. This problem can be overcome by incorporating measures of genetic relatedness, population structure, or both into mixed models (Kang et al., 2008; Bradbury et al., 2011).

Our long-term goal is to add to the catalog of useful rust resistance genes from wild wheat relatives for cultivated wheat improvement. The objectives of this study were to: (1) perform comprehensive phenotyping of the ALDIVCO by expanding evaluations for resistance to additional races of the stem rust, leaf rust, and stripe rust pathogens; and (2) utilize GWAS to identify loci influencing rust resistance present in the panel. In this investigation, we found that *Ae. longissima* is a rich source of diverse resistance to the rust pathogens of wheat, and we report genetic markers significantly associated with rust resistance to multiple rust pathogens and pathogen races.

## 2 Materials and methods

### 2.1 Plant materials

The *Ae. longissima* Diversity Collection (ALDIVCO, n = 433) was assembled and utilized for rust phenotyping by Huang et al. (2018). This panel consists of 433 accessions: 424 from Israel, four from Jordan, and five from unknown sites. Most (411) of the ALDIVCO accessions were provided by the Harold and Adele Lieberman Germplasm Bank in the Institute for Cereal Crops Research (ICCR) at Tel Aviv University (Tel Aviv, Israel), with remaining accessions donated by the Leibniz-Institut für Pflanzengenetik und Kulturpflanzenforschung (IPK) in Gatersleben, Germany. A subset of this collection (n = 295-380) was used for all rust evaluations (**Supplementary Table S1**) in Huang et al. (2018) and in the current study.

Most of the accessions phenotyped by Huang et al. (2018) were selfed for one to four generations. In this study, several additional generations of selfing (up to six) were done on the panel before phenotyping. These additional selfing generations served to increase homozygosity and generate additional seed stocks for further evaluations. Poor plant growth and issues with flowering and sterility resulted in low seed stocks for several accessions; thus, the number of accessions included in any one rust evaluation ranged from 295-380 accessions.

Susceptible controls were included in each experiment to assess the infection level and virulence of the pathogen races. Wheat cultivars McNair 701 (PI 518817), Thatcher (PI 168659), and Morocco (PI 431591) were the susceptible controls for the stem rust, leaf rust, and stripe rust evaluations, respectively. Respective wheat differential lines were included in all rust phenotyping experiments to confirm the identity of pathogen races utilized (Roelfs and Martens, 1988; Long and Kolmer, 1989; Jin et al., 2008; Wan and Chen, 2014). The respective resistant differential lines to each race served as resistant controls.

### 2.2 Rust pathogen isolates

Huang et al. (2018) evaluated the ALDIVCO for resistance to two races of *Pgt* (TTTTF and TTKSK), two races of *Pt* (BBBDB and THBJG), and one race of *Pst* (PSTv-37). The phenotypic data generated by Huang et al. (2018) were utilized in this study for GWAS. Two additional races for each of the three rust pathogens were selected for evaluation in this study (**Table 1**). Race TTRTF (isolate 14GEO189-1-B) of *Pgt* was selected because it is widely virulent and is becoming one of the most widely distributed races in Europe and the Middle East (Olivera et al., 2019; Omrani and Roohparvar, 2021). In contrast, *Pgt* race QFCSC (isolate 06ND76C) was selected because it has a narrower virulence spectrum but is also one of the predominant races in the United States (Jin et al., 2014).

**Table 1:** Race, isolate, virulence phenotype, and source of wheat rust pathogens used to evaluate resistance in *Aegilops longissima*.

Pathogen	Race^a^	Isolate	Virulence/avirulence formula^b^	Source
*Puccinia graminis* f. sp. *tritici*	TTTTF*	02MN84A-1-2	5, 6, 7b, 8a, 9a, 9b, 9d, 9e, 9g, 10, 11, 17, 21, 30, 36, 38, McN, Tmp/24, 31	Y. Jin (USDA-ARS Cereal Disease Laboratory. St. Paul, MN)
*P. graminis* f. sp. *tritici*	TTKSK*	04KEN156/04	5, 6, 7b, 8a, 9a, 9b, 9d, 9e, 9g, 10, 11, 17, 21, 30, 31, 38, McN/24, 36, Tmp	Y. Jin (USDA-ARS Cereal Disease Laboratory. St. Paul, MN)
*P. graminis* f. sp. *tritici*	TTRTF	14GEO189-1-B	5, 6, 7b, 8a, 9a, 9b, 9d, 9e, 9g, 10, 11, 21, 36, McN, Tmp/17, 30, 24, 31, 38	Y. Jin (USDA-ARS Cereal Disease Laboratory. St. Paul, MN)
*P. graminis* f. sp. *tritici*	QFCSC	06ND76C	5, 8a, 9a, 9d, 9g, 10, 17, 21, 35, McN/6, 7b, 9e, 9b, 11, 22, 24, 31, 30, 36, 38, Tmp	Y. Jin (USDA-ARS Cereal Disease Laboratory. St. Paul, MN)
*P. triticina*	THBJG*	99ND588DLL	1, 2a, 2c, 3a, 16, 26, 10, 14a, 18/9, 24, 3ka, 11, 17, 30, B	J. Kolmer (USDA-ARS Cereal Disease Laboratory. St. Paul, MN)
*P. triticina*	BBBDB*	JAK	14a/1, 2a, 2c, 3a, 9, 16, 24, 26, 3ka, 11, 17, 30, B, 10, 18	J. Kolmer (USDA-ARS Cereal Disease Laboratory. St. Paul, MN)
*P. triticina*	BBBQD	CA 1.2	B, 10, 39/1, 2a, 2c, 3, 9, 16, 24, 26, 3ka, 11, 17, 30, 14a, 18, 21, 28, 42	J. Kolmer (USDA-ARS Cereal Disease Laboratory. St. Paul, MN)
*P. triticina*	TBBGS	14 US 154-1	1, 2a, 2c, 3, 10, 21, 28, 39/9, 16, 24, 26, 3ka, 11, 17, 30, B, 14a, 18, 42	J. Kolmer (USDA-ARS Cereal Disease Laboratory. St. Paul, MN)
*P. striiformis* f. sp. *tritici*	PSTv37*	10-106	6, 7, 8, 9, 17, 27, 43, 44, Tr1, Exp2/1, 5, 10, 15, 24, 32, SP, Tye	X. Chen (USDA-ARS Wheat Genetics, Physiology, Quality, and Disease Research Unit and Department of Plant Pathology, Washington State University, Pullman, WA)
*P. striiformis* f. sp. *tritici*	PSTv40	10-391	6, 7, 8, 9, 10, 24, 27, 32, 43, 44, Tr1, Exp2/1, 5, 15, 17, SP, Tye	X. Chen (USDA-ARS Wheat Genetics, Physiology, Quality, and Disease Research Unit and Department of Plant Pathology, Washington State University, Pullman, WA)
*P. striiformis* f. sp. *tritici*	PSTv218	2008-012	6, 7, 8, 9, 10, 24, 32, 43, 44, SP, Tr1, Exp2/1, 5, 15, 17, 27, Tye	X. Chen (USDA-ARS Wheat Genetics, Physiology, Quality, and Disease Research Unit and Department of Plant Pathology, Washington State University, Pullman, WA)

a Races of the pathogens were characterized on the respective wheat differential host sets for stem rust (Roelfs and Martens, 1988; Jin et al., 2008), leaf rust (Long and Kolmer, 1989), and stripe rust (Wan and Chen, 2014).

b The virulence/avirulence formulae represent the resistance genes of the differential wheat genotypes for which the pathogen races possess virulence or avirulence.

* Races previously evaluated by Huang et al. (2018).

Race BBBQD (isolate CA 1.2) of *Pt* was selected because it is representative of a worldwide group of isolates that have high virulence to durum wheat (*Triticum turgidum* ssp. *durum*) but is generally avirulent to most bread wheats and most *Lr* genes (Kolmer, 2015; Kolmer and Fajolu, 2022). In contrast, *Pt* race TBBGS (isolate 14 US 154-1) was selected because it has a wide virulence spectrum. Of note, this race is virulent to *Lr21*, a resistance gene commonly present in some hard red spring wheat cultivars and virulent to *Lr39*, which is very common in hard red winter wheat cultivars. TBBGS is commonly found in the northern Great Plains of the United States (Kolmer and Fajolu, 2022).

*Pst* races PSTv218 (isolate 2008-012) and PSTv40 (isolate 10-391) were selected because they both have relatively wide, but differing virulence spectra (i.e., both are virulent on 12 out of 18 *Yr* genes in the Avocet differential set; Wan et al., 2017), allowing for identification of accessions with broadly effective resistance to stripe rust.

The virulence phenotypes for isolates of all three pathogens were verified on their respective differential sets (Roelfs and Martens, 1988; Long and Kolmer, 1989; Jin et al., 2008; Wan and Chen, 2014).

### 2.3 Plant growth conditions

As a wild grass, *Ae. longissima* seeds often require cold stratification to break dormancy. To provide the required cold period, seeds were either placed on moistened filter paper in 9-cm petri dishes and incubated at 4°C for five days before being transferred to growing medium or were directly planted into moistened growing medium and incubated at 10°C for seven days. For the rust phenotyping experiments, three seeds per accession were sown plastic cones (3.8 cm diameter x 21 cm depth) held in racks of 98 units or peat pots (7 x 7 x 9 cm; l x w x h) containing a mixture of 50:50 (by volume) steam-sterilized native soil: half plant growth medium (Sunshine MVP mix; Sungrow Horticulture Distributors, Quincy, MI).

Plants for assessments to all *Pt* races, all *Pst* races, and *Pgt* races TTTTF and QFCSC were grown in a greenhouse at the University of Minnesota Plant Growth Facility (St. Paul, MN) under a temperature regime of 25/17°C (day/night) and 16-hour photoperiod (400-W high pressure sodium lamps, emitting a minimum of 300 µmol photons s^-1^ m^-2^) until inoculation. Plants for assessments to *Pgt* races TTKSK and TTRTF were grown in the Minnesota Agricultural Experiment Station/Minnesota Department of Agriculture Biosafety Level-3 (BSL-3) Containment Facility (St. Paul) in a greenhouse under a temperature regime of 22/19°C (day/night) and 14-hour photoperiod (400-W high pressure sodium lamps, emitting a minimum of 300 µmol photons s^-1^ m^-2^).

During the course of all rust phenotyping experiments, four fertilizer applications were given to all plants: two at planting (0.3 g/pot slow-release Osmocote 14-14-14 and approximately 40 g/L at 1/16 dilution of Peter’s Dark Weather 15-0-15; Scott’s Company, Marysville, OH) and two additional applications (approximately 40 g/L at 1/16 dilution of Peter’s 20-10-20; Scott’s Company) at one-week intervals until the plants were scored.

### 2.4 Seedling resistance assessments

Prior to inoculation, urediniospores of the rust pathogens were removed from a -80°C freezer, heat-shocked in a 45°C water bath for 15 min, then rehydrated in an 80% relative humidity chamber for 1-16 hr and then suspended in a lightweight mineral oil (Soltrol 170; Phillips Petroleum, Bartlesville, OK). The suspension was applied to the plants using custom atomizers pressured at 25-30 kPa (Tallgrass Solutions, Inc., Manhattan, KS), delivering approximately 0.04 mg, 0.04 mg, and 0.09 mg urediniospores per plant for *Pgt, Pt*, and *Pst*, respectively. The different concentrations of inoculum used reflect the varied infectivity rates of each pathogen. Inoculated plants were placed in front of fans for five minutes to hasten evaporation of the oil carrier from leaf surfaces. The plants were left in open air for an additional 90 minutes to ensure oil evaporation before being placed in chambers to induce rust infection.

Plants inoculated with *Pgt* and *Pt* were placed in mist chambers and those inoculated with *Pst* were placed in dew chambers under the conditions previously described (Huang et al., 2018). After the induction of infection, plants were returned to their respective growing conditions as described above, with the exception of those inoculated with *Pst*, which were transferred to a growth chamber with a temperature cycle changing from 18°C to 16°C at 2200 h, then to 14°C at 2300 h, and back to 18°C at 0600 h with a 16 hour photoperiod (400-W high pressure sodium lamps, emitting a minimum of 300 µmol photons s^-1^ m^-2^).

Stem and leaf rust infection types (ITs) were assessed 12-14 days post-inoculation based on 0-4 rating scales (Stakman et al., 1962; Roelfs and Martens, 1988; Long and Kolmer, 1989). Stripe rust ITs were assessed 19 days post-inoculation based on a 0-9 rating scale (Wan and Chen, 2014). Disease assessments were made on the ALDIVCO in at least two separate experiments conducted in a completely randomized design with repeated controls. Accessions giving highly variable reactions across replicates were screened an additional time.

For GWAS, the categorical phenotype data for the stem and leaf rust experiments were converted to a 0-9 linear disease scale as described previously (Zhang et al., 2014). Simple ITs were converted as follows: 0/;, 1-, 1, 1+, 2-, 2, 2 + 3-, 3, and 3+ were coded as 0, 1, 2, 3, 4, 5, 6, 7, 8, and 9 respectively. For more complex reactions, such as “;12” (meaning “;” or “fleck” was the most prevalent IT, 1 was the second most prevalent IT, and 2 was the least prevalent IT), only the first and second listed ITs (i.e., the most prevalent and second most prevalent) were converted to the linear scale and then averaged, with the first IT being double weighted. Then the linearized scores from all replications were averaged. Generally, categorical ITs are divided into two general reaction classes: resistant (ITs ranging from 0 to 2+ for *Pgt* and *Pt* or 0-6 for *Pst*) and susceptible (ITs ranging from 3- to 4 for *Pgt* and *Pt* or 7-9 for *Pst*). Accessions were classified as heterogeneous if they included both resistant and susceptible plants, either within the same experiment or across repeated experiments. Data from heterogeneous accessions in experiments with *Pgt* and *Pt* were not included in further analyses. Due to more quantitative nature of disease severity ratings, data from heterogeneous accessions in *Pst* experiments were not retained for further analyses only if the absolute value of the difference between the ITs of two or more replications was greater than two units on the linearized scale.

### 2.5 Genotyping

Genome-wide markers were obtained using the genotyping-by-sequencing (GBS) approach (Poland et al., 2012). DNA was extracted from seedling tissue by the University of Minnesota Genomics Center (UMGC) using their standard plant 2% CTAB protocol. GBS libraries were created using the *Pst1-Msp1* restriction enzymes. The samples were pooled together at 96-plex to create pooled libraries, which were then sequenced on Illumina NextSeq 500. A total of 384 accessions were genotyped.

SNP calling performed with the TASSEL v5 GBSv2 pipeline (Glaubitz et al., 2014) using 64 k-mer length and minimum k-mer count of 20. Reads were aligned to the reference chromosome-scale sequence assembly of *Ae. longissima* (accession AEG-6782-2, Avni et al., 2022) using the aln method of the Burrows-Wheeler aligner (bwa) (Li and Durbin, 2009). Raw SNP data generated from the GBSv2 pipeline were then filtered to remove taxa with >90% missing data (n = 3), sites with genotype quality<30, and sites with minor allele frequency (MAF) ≤ 0.01, leaving 283,104 sites. Additional filtering steps were performed including selecting biallelic SNPs with missing data ≤ 20% and heterozygosity ≤ 5%. After filtering, 141,218 SNP markers remained for the remaining 381 accessions. Genotypic data for the ALDIVCO is deposited in the Triticeae Toolbox (https://wheat.triticeaetoolbox.org/breeders/trial/9452)

### 2.6 Linkage disequilibrium, population structure, and genetic distance

Full matrices of pairwise comparison between markers (with< 20% missing data) for each chromosome was used to characterize the linkage disequilibrium (LD, as *r^2^*) decay in the ALDIVCO using TASSEL v5. These LD estimates were plotted against physical distance. Locally weighted scatter plot smoother (LOWESS) was run in JMP (JMP, Version 14.3.0, SAS Institute Inc., Cary, NC) to visualize the change of LD with physical distance (**Supplementary Figure S1**). Markers that were in perfect LD (*r^2 ^* = 1) with an adjacent marker (n = 15,875) were removed, leaving 125,343 sites for association analyses. Average distance between adjacent markers and average adjacent marker LD along each chromosome were plotted as heatmaps (**Supplementary Figure S2**).

**Figure 1:**
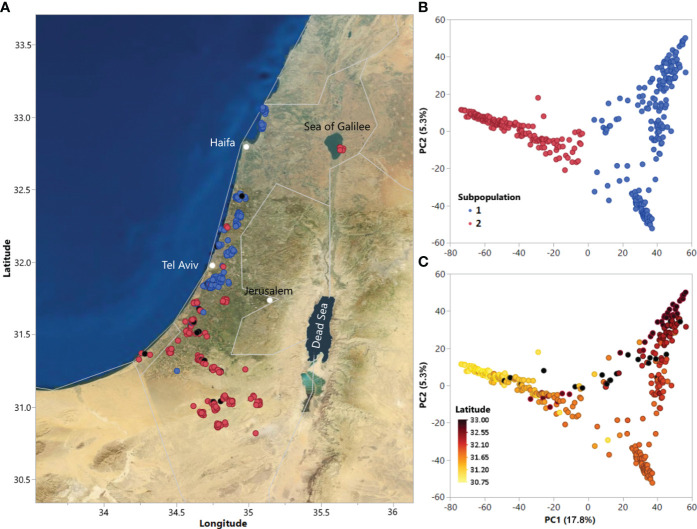
**(A)** Geographic distribution of accessions in the ALDIVCO and **(B)** Principal component analysis (PCA) of 378 accessions of the ALDIVCO using 125,343 markers. Two subpopulations were inferred by *K*-means clustering. **(C)** PCA of the ALDIVCO colored according to latitude.

Principal component analysis (PCA) was employed to detect population structure using TASSEL v5. The final set of markers used to determine covariates for GWAS and the first two principal components were plotted to visualize population structure (**Figure 1B**). In addition to PCA, characterization of subpopulations was performed with *K*-means clustering using the R package factoextra (Kassambara and Mundt, 2020), and the optimal number of subpopulations for analysis was determined using the R package NbClust (Charrad et al., 2014). Hierarchical clustering utilizing Ward’s minimum variance method on the genetic distance matrix was also performed in JMP to examine relatedness among accessions (**Supplementary Figure S2**).

### 2.7 Phenotypic data analysis

**Figure 2:**
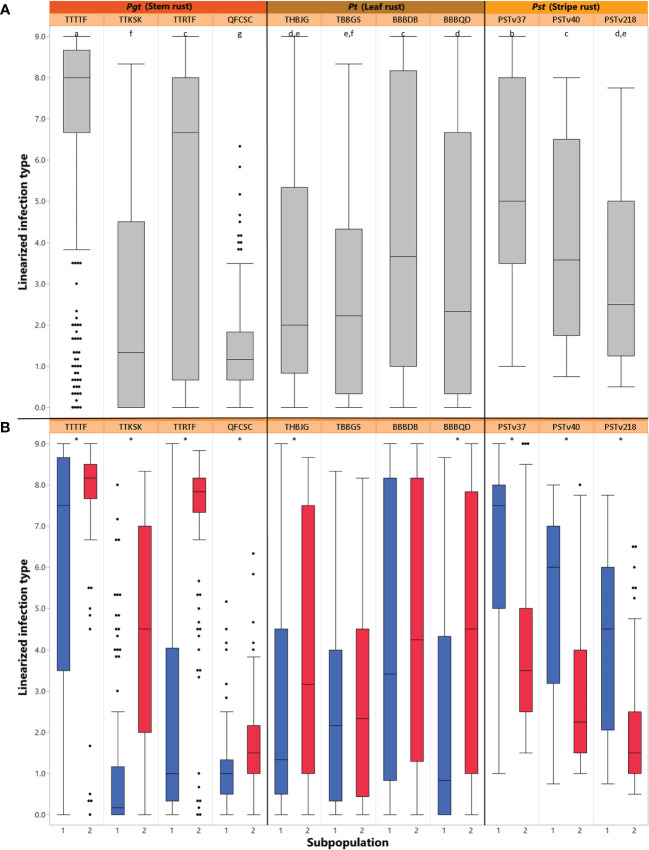
**(A)** Box plot for distributions of mean linearized seedling infection types (ITs) for four races of *Puccinia graminis f* sp. *tritici*, four races of *Puccinia triticina*, and three races of *Puccinia striiformis f.* sp. *tritici*. Solid horizontal lines show medians. The top and bottom box edges show the 25th to 75th percentiles of total data. Different letters above box plots indicate significant differences between means (*p*< 0.05, Tukey HSD). **(B)** Box plot for distributions of mean linearized seedling infection types (ITs) for four races of *Pgt*, four races of *Pt*, and three races of *Pst* split between two subpopulations of the ALDIVCO as inferred by *K*-means clustering. Asterisks above the plots indicate a significant difference between subpopulation means (*p*< 0.05, two-sample t-test).


Mean linearized ITs for the ALDIVCO to all rust pathogen isolates were visualized as histograms and boxplots. To improve normality of the rust phenotyping data for association analyses, the mean linearized ITs were subjected to log_10_ + 1 transformations. The Shapiro-Wilk test was performed in JMP to check for normality of the transformed and untransformed mean ITs. Pearson correlation coefficients for pairwise comparisons between rust races were calculated using mean ITs in R (**Supplementary Figure S4**). Comparison of subpopulation phenotype means was performed for each rust race using two-sample t-tests with α = 0.05 (**Figure 2B**). Comparison of phenotype means across races was performed using ANOVA and Tukey-Kramer HSD with α = 0.05 (**Figure 2A**). Additionally, ANOVA was performed for all experiments using the mean linearized ITs and variance components were estimated from the expected mean squares of the ANOVA. Reliability (*i^2^*) (also referred to as broad-sense heritability, *H^2^*) for resistance to all races in the ALDIVCO was estimated on an entry mean basis using the equation:

$$i^{2}=\;\frac{\sigma_{g}^{2}}{\sigma_{g}^{2}+\left(\frac{\sigma_{e}^{2}}{n}\right)}$$

where $\sigma_{g}^{2}$ is the estimated genetic variance, $\sigma_{e}^{2}$ is the estimated error variance, and *n* is the number of replications.

### 2.8 GWAS analyses

Markers associated with resistance to all races of *Pt*, *Pgt*, and *Pst* were identified using three different GWAS models: (1) A mixed linear model (MLM), (2) Fixed and random model Circulating Probability Unification (FarmCPU; Liu et al., 2016) and (3) Bayesian-information and Linkage-disequilibrium Iteratively Nested Keyway (BLINK; Huang et al., 2019). The MLM accounted for population structure (*Q*) + kinship (*K*). The model is described as


y= μ+Wm+Qv+Zu+ ε

where **y** is the vector of the resistance phenotype mean across experiments, µ is the population mean, **m** is the vector of fixed SNP effects, **v** is the vector of fixed population effect, **u** is the vector of random genetic background effect for each accession, and **ϵ** is the vector of residuals. W, Q, and Z are incidence matrices. The parameter **u** is distributed as $N\left(0,K\sigma_{g}^{2}\right)$ where *K* is the kinship matrix and $\sigma_{g}^{2}$ is the genetic variance. The parameter **ϵ** is distributed as $N\left(0,I\sigma_{\varepsilon }^{2}\right)$ , where *I* is the identity matrix and $\sigma_{\varepsilon }^{2}$ is the error variance. The first two principal components from the PCA and the additive relationship matrix were used to account for population structure and genetic relatedness. FarmCPU and BLINK models were also used to overcome model overfitting problems of stepwise regression. FarmCPU uses an algorithm that considers the confounding problem between covariates and test marker by iterating between both a Fixed Effect Model (FEM) and a Random Effect Model (REM). BLINK replaces restricted maximum likelihood (REML) in FarmCPU’s REM with Baysian information criteria in a FEM and uses LD information to eliminate the restricting assumption that causal genes are evenly distributed across the genome. The first two principal components were fitted as covariates in both models to reduce false positives due to population structure. The models were implemented in GAPIT v3 package in R (Wang and Zhang, 2021). Multiple testing comparisons for GWAS were taken into account using the Bonferroni correction (α = 0.05), leading to an experiment-wide significance threshold of 0.05/125,343 markers = 3.99 × 10^-7^ or –log_10_3.99 × 10^-7 ^= 6.40. QQ plots were utilized to evaluate the fit of the three different models (**Supplementary Figure S5**).

GWAS using all three models was also performed on each of the two subpopulations identified via *K*-means clustering independently for all races of the rust pathogens evaluated. Each genotypic matrix for the two subpopulations was filtered again to remove markers with minor allele frequency (MAF) ≤ 0.01 and missing data ≥ 20%, leaving 109,110 markers for the 204 accessions in subpopulation 1 and 84,027 markers for the 174 accessions in subpopulation 2. Therefore, the significance threshold (using Bonferroni, α = 0.05) for each subpopulation was 0.05/109,110 markers = 4.58 × 10^-7^ or –log_10_4.58 × 10^-7 ^= 6.34 for subpopulation 1 and 0.05/84,027 markers = 5.95 × 10^-7^ or –log_10_5.95 × 10^-7 ^= 6.23 for subpopulation 2.

### 2.9 Candidate gene identification

Putative candidate genes were identified by inspecting the reference genome assembly of *Ae. longissima* (accession AEG-6782-2, Avni et al., 2022) flanking key loci identified via GWAS. This 6.70 Gbp assembly has an estimated 97.5% genome annotation completeness based on BUSCO analysis (Simão et al., 2015). Flanking regions of one Mbp in either direction of key loci were captured using the UCSC Genome Browser (Kent et al., 2002). Key loci included those that were detected at least three times, meaning they were identified by all three GWAS models or were significant across multiple rust races and/or subpopulations. Candidate genes near key loci identified on scaffolds unassigned to chromosome assemblies (“chromosome UN”) were restricted to gene annotations on the same contig as the marker.

## 3 Results

### 3.1 Genotypic analyses, LD, population structure, and genetic relatedness

Upon filtering of the markers generated by GBS and subsequent alignment to the *Ae. longissima* reference genome assembly, 141,218 SNP markers were identified in the subset (n = 381) of the ALDIVCO and used for further analysis. Removal of adjacent markers in perfect LD (*r^2 ^* = 1) resulted in 125,343 SNPs used in the final analyses. The percentage of missing genotypic data in this set was 42.0%, with an average minor allele frequency of 0.21. The average adjacent marker LD (estimated as *r^2^*) for individual chromosomes ranged from 0.15 to 0.19 and was 0.17 across all chromosomes (**Supplemental Table S4**). Using a sliding window of 25 markers, the overall average marker LD across the genome was 0.14.

Using full matrices of pairwise comparison between markers (with< 20% missing data) for each chromosome, the marker LD decayed rapidly in the first 20 Mbp, then tended to level off at around *r^2^* of 0.04-0.05 (**Supplementary Figure S1**). For chromosomes 3S, 4S, and 7S, a slight increase in LD was observed from approximately 20 Mbp to 440, 260, and 280 Mbp respectively. A pattern of longer distance between available markers near the central regions of chromosomes was observed (**Supplementary Figure S2A**). Average adjacent marker LD was also high in the central regions of the chromosomes, with the exception of chromosomes 5S (**Supplementary Figure S2B**).

Using the genotypic matrix of 125,343 markers across the genome, PCA was used for estimating genetic relatedness of the accessions (**Figure 1A**). Cluster analysis using *K*-means clustering identified two subpopulations, represented on the PC plot and map of accession collection sites (**Figures 1A, B**). PC1 and PC2 explained 17.8 and 5.3% of the variability, respectively. Subpopulation 1 (n = 204) mostly included accessions along the coastal plain north of latitude 31.84, while subpopulation 2 (n = 174) mostly included accessions from south of latitude 31.84, extending southeast into the Negev desert, plus a geographically separated group of seven accessions on the eastern shore of the Sea of Galilee. In general, genetic relatedness of accessions followed a latitudinal gradient from north to south (**Figure 1C**). Hierarchical clustering utilizing Ward’s minimum variance method on the genetic distance matrix revealed a similar pattern of relatedness among the accessions (**Supplementary Figure S3**).

### 3.2 Phenotypic analyses

Uniform infection was obtained on all susceptible controls and *Ae. longissima* accessions in all experiments, with the exception of one experiment for *Pst* race PSTv40 where infection was uncharacteristically low, and thus an additional experiment was conducted for accessions with infection types diverging by at least 2.5 units. The complete set of raw ITs for all experiments and average linearized ITs are given in **Supplementary Table S1**.

A wide range of variation was found among all accessions in response to all pathogen races evaluated, with the exception of *Pgt* race QFCSC, for which all accessions displayed resistant responses (ITs of 0 to 2+) (**Table 2**). The percentage of resistant accessions varied widely to the other three *Pgt* races evaluated: over 80% were resistant to TTKSK, compared with 43.7% to TTRTF, and only 18.5% to TTTTF. The percentage of heterogeneous accessions (i.e., those displaying both resistant and susceptible plants) for the three virulent races ranged from 4.9% for TTKSK to 17.6% for TTTTF. The percentage of resistant accessions was more comparable for races of *Pt* with 52.2% for BBBDB, 61.4% for BBBQD, 66.0% for THBJG, and 68.5% for TBBGS (**Table 2**). The percentage of heterogenous accessions for *Pt* ranged from 13.7% for race THBJG to 24.8% for race TBBGS. The percentage of resistant accessions was similar for *Pst* races PSTv37 (49.5%) and PSTv40 (53.7%) but was notably higher for race PSTv218 (79.0%) (**Table 2**). The percentage of heterogeneous accessions for *Pst* ranged from 7.1% for race PSTv37 to 31.5% for race PSTv40.

**Table 2:** Number and percentage of *Aegilops longissima* accessions exhibiting resistant, susceptible, and heterogeneous reactions to three wheat rust pathogens at the seedling stage.

Pathogen	Race	Resistant^a^	Susceptible^b^	Heterogeneous^c^	Total
*Puccinia graminis* f. sp. *tritici*	TTTTF*	64 (18.5%)	221 (63.9%)	61 (17.6%)	346
*P. graminis* f. sp. *tritici*	TTKSK*	249 (80.6%)	45 (14.6%)	15 (4.9%)	309
*P. graminis* f. sp. *tritici*	TTRTF	155 (43.7%)	152 (42.8%)	48 (13.5%)	355
*P. graminis* f. sp. *tritici*	QFCSC	354 (99.7%)	0 (0.0%)	1 (0.3%)	355
*P. triticina*	THBJG*	246 (66.0%)	76 (20.4%)	51 (13.7%)	373
*P. triticina*	BBBDB*	179 (52.2%)	102 (29.7%)	62 (18.1%)	343
*P. triticina*	BBBQD	218 (61.4%)	68 (19.2%)	69 (19.4%)	355
*P. triticina*	TBBGS	243 (68.5%)	24 (6.8%)	88 (24.8%)	355
*P. striiformis* f. sp. *tritici*	PSTv37*	188 (49.5%)	165 (43.4%)	27 (7.1%)	380
*P. striiformis* f. sp. *tritici*	PSTv40	191 (53.7%)	53 (14.9%)	112 (31.5%)	356
*P. striiformis* f. sp. *tritici*	PSTv218	282 (79.0%)	19 (5.3%)	56 (15.7%)	357

a Number of resistant (those exhibiting infection types [ITs] of 0 to 2+ for stem and leaf rust, and 0 to 6 for stripe rust) accessions observed to each pathogen race. Frequency is given in parentheses.

b Number of susceptible (those exhibiting ITs of 3- to 4 for stem and leaf rust, and 7 to 9 for stripe rust) accessions observed to each pathogen race. Frequency is given in parentheses.

c Heterogeneous indicates that individual accessions exhibited a mixture of distinctly resistant and susceptible plants.

*Races previously evaluated by Huang et al. (2018).

Forty-one (out of 404 total evaluated against at least one pathogen race; 10.1%) accessions were resistant to all four *Pgt* races. Eighty-nine (22.0%) accessions were resistant to three of the four races, 137 (33.9%) were resistant to two of the four races, and 117 (29.0%) were resistant to just one of the four races. One hundred and four (25.7%) accessions were resistant to all four *Pt* races, 81 (20.0%) were resistant to three of the four races, 79 (19.6%) were resistant to two of the four races, and 69 (17.1%) were resistant to just one of the four races. One hundred and twenty-one (30.0%) accessions were resistant to all three *Pst* races, 99 (24.5%) were resistant to two of the three races, and 100 (24.8%) were resistant to just one of the three races. There were 295 (73%) accessions that were resistant to at least five of the rust pathogen races evaluated (not all accessions were evaluated against all races; **Table S1**). Eleven accessions were resistant to 10 of the 11 pathogen races evaluated, although seven of these were susceptible or had heterogeneous reactions to *Pgt* race TTTTF. Two ALDIVCO accessions (AEG-1471-15 and AEG-2974-0) were resistant to all pathogen races evaluated, both belonging to subpopulation 2.

Reliability (often referred to as broad-sense heritability) estimates were calculated for the reactions of the ALDIVCO to all rust races evaluated (**Supplementary Table S4**). Reliabilities were high for all races, ranging from 0.76 for *Pst* race PSTv40 to 0.95 for *Pgt* race TTRTF. Mean linearized ITs of the ALDVICO to all pathogen races of the same species were positively correlated with one another and highly significant (P< 0.00001) (**Supplementary Figure S4**). Mean linearized ITs of *Pt* races were typically not correlated, or weakly positively correlated, with those of *Pgt* races and *Pst* races. In contrast, mean linearized ITs of all *Pgt* races were negatively correlated with those of *Pst* races (P< 0.01). The highest positive *r* ^2^ value (0.76) was found between *Pst* races PSTv40 and PSTv218, while the most negative correlation (*r* ^2^ = -0.42) was found between *Pgt* TTRTF and *Pst* race PSTv218.

The median linearized IT for TTTTF was 8.0, the highest of all races, followed by TTRTF with 6.3 (**Figure 2A**). By separating out the average linear ITs by subpopulation cluster, it was revealed that for all races of *Pgt* and *Pt*, subpopulation 1 had lower means than subpopulation 2 (**Figure 2B**). However, this pattern was reversed for all races of *Pst*, where subpopulation 2 had lower means than subpopulation 1. For all pathogen races evaluated, with the exception of TBBGS and BBBDB of *Pt*, the two subpopulations had significantly different means as revealed by two-sample t-tests (α = 0.05; **Figure 2B**).

The mean linearized ITs for each race were subjected to log_10 + _1 transformations and then the Shapiro-Wilk test was performed to check for normality of the transformed and untransformed data. For all races except *Pgt* races TTTTF and TTRTF and *Pst* races PSTv37 and PSTv40, the transformed data were more normal (i.e., had a larger value of the test statistic, *W*; data not shown). However, the slight increase in normality achieved was deemed insufficient to justify using transformed means for association analyses to avoid unintended biasing of the data.

### 3.3 GWAS for rust resistance

Genome-wide association mapping for seedling resistance to each of the 11 pathogen races was conducted using 381 *Ae. longissima* accessions and 125,343 SNP markers, via three models in the GAPIT R package: MLM, FarmCPU, and BLINK. GWAS using all three models was also performed on each of the two identified subpopulations independently for all races of rust pathogens evaluated (using 109,110 markers and 84,027 markers for the 204 and 174 accessions in subpopulation 1 and 2, respectively).

The single-variant MLM tended to have a worse fit than either the FarmCPU or BLINK models, as shown by QQ plots with slightly inflated or deflated *p*-values, depending on the race and/or subpopulation under investigation (**Supplementary Figure S5**). For example, in subpopulation 1, the MLM for *Pgt* race QFCSC showed inflated *p*-values, and a similar trend was observed in subpopulation 2 for *Pgt* race TTTTF, TTRTF, and QFCSC.

**Figure 3:**
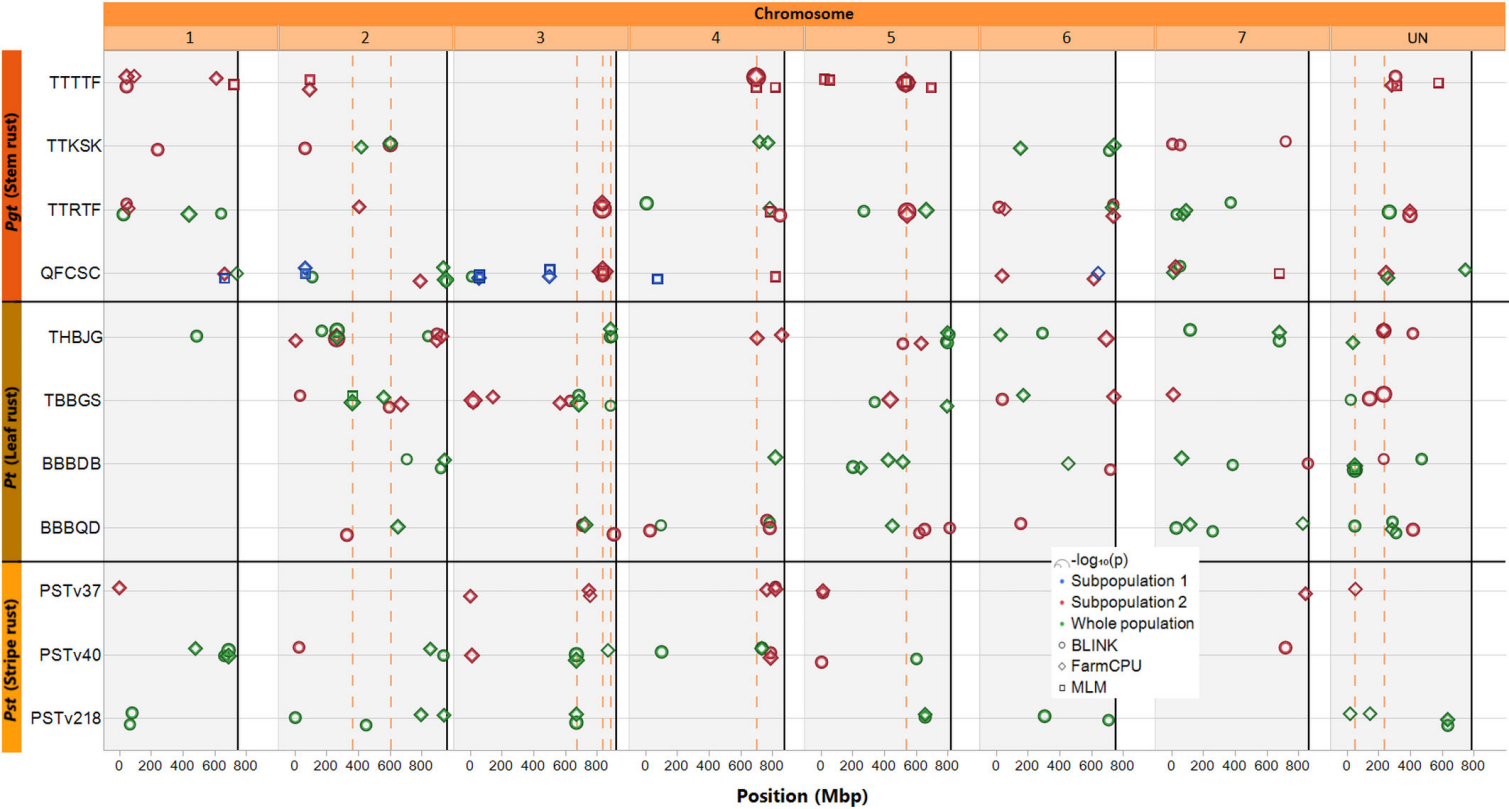
Map of markers significantly associated with rust seedling infection types to four races of *Puccinia graminis* f. sp. *tritici*, four races of *Puccinia triticina*, and three races of *Puccinia striiformis* f. sp. *tritici*. Solid black vertical lines indicate the end of each chromosome. Circle symbols indicate markers associated by the BLINK algorithm, diamond symbols indicate markers associated by the FarmCPU algorithm, and square symbols indicate markers associated by a mixed linear model (MLM), all using the first two principal components as covariates. Only markers with *p*-values meeting a Bonferroni corrected (α = 0.05; 0.05/total number of markers) threshold are displayed. Green symbols indicate significant markers identified using the entire population of accessions screened, while blue and red symbols indicate markers identified by the models run independently in either subpopulation 1 or 2, respectively (as assigned via *K*-means clustering). The symbols are scaled by –log_10_(*p*), such that larger symbols indicate higher significance. The dashed orange vertical lines indicate markers that were detected at least three times (either by all three models, or in multiple population GWAS, and/or for resistance to multiple pathogen races).

Utilizing the whole population for GWAS, significant marker-trait associations (MTAs) were identified for nine of the eleven pathogen race combinations: *Pgt* TTKSK, TTRTF, and QFCSC; *Pt* THBJG, TBBGS, BBBDB, and BBBQD; and *Pst* PSTv40 and PSTv218 (**Supplementary Table S2**, **Figure 3**). When GWAS was performed in subpopulation 2, significant MTAs were detected for ten of the pathogen race combinations with the exception of *Pst* PSTv218. In subpopulation 1, significant MTAs were detected for only one pathogen race, *Pgt* QFCSC. In total, 195 significant MTAs for seedling resistance were detected by at least one model for the whole population and/or by at least one model for either of the subpopulations independently (**Supplementary Table 2**, **Figure 3**).

Three markers were significantly associated with resistance to two pathogen races: one (S3S_669959829) on chromosome 3S identified for resistance to races PSTv40 and PSTv218 in the whole population by both FarmCPU and BLINK, a second (S3S_885155266) on chromosome 3S identified in the whole population by FarmCPU and BLINK for *Pt* THBJG and by BLINK for *Pt* TBBGS, and the third (SUN_49934383) on a scaffold unassigned to any chromosome (chromosome UN) identified for resistance to *Pt* BBBDB (by all three models) *Pt* BBBQD (BLINK model only) in the whole population. One marker (SUN_232304889) on chromosome UN was significantly associated with resistance to three *Pt* races: THBJG (detected by FarmCPU and BLINK models), TBBGS (detected by BLINK), and BBBDB (detected by BLINK) in subpopulation 2. In subpopulation 2, three SNP markers were identified as significant by all three models: one for *Pgt* QFCSC on chromosome 3S (S3S_835968584) and two for *Pgt* TTTTF, one on chromosome 4S and the other on chromosome 5S (S4S_697635226, S5S_535624330). One marker (S2S_363051049) on chromosome 2S identified for resistance to *Pt* TBBGS in the whole population was identified by all three models in the whole population. Lastly, one marker (S2S_602532465) on chromosome 2S was associated with resistance to *Pgt* TTKSK by FarmCPU and BLINK in the whole population and by BLINK in subpopulation 2.

These nine markers were detected at least three times in the GWAS analysis (**Table 3**), i.e., by all three models in either the whole population or subpopulation 2, by two models in the whole population and one in subpopulation two or across multiple races with the same and/or different models.

**Table 3:** Nine key significant marker loci associated with seedling resistance in the ALDIVCO to one race of *Puccinia graminis* f. sp. *tritici*, four races of *P*. *triticina*, and two races of *P. striiformis* f. sp. *tritici*.

Pathogen	Race(s)	Models	Population(s) identified in^a^	Marker ID	Chromo-some	Position (Mbp)	*p*-value	n obs^b^	MAF^c^	effect^d^	Putative candidate gene(s) function^e^
*Pt*	TBBGS	MLM	Whole	S2S_363051049	2S	363.05	2.63E-07	264	0.47	1.48	Cyclic nucleotide-gated cation channel α-3
		FarmCPU					4.64E-13	264	0.47	1.22	
		BLINK					9.48E-09	264	0.47	1.1	
*Pgt*	TTKSK	FarmCPU	Whole	S2S_602532465	2S	602.53	2.93E-09	278	0.28	0.72	F-box protein family-like, F-box family protein, LURP-one-like protein
		BLINK					3.41E-07	278	0.28	0.82	
		BLINK	Subpop 2				2.96E-15	120	0.43	1.28	
*Pst*	PSTv40	FarmCPU	Whole	S3S_669959829	3S	669.96	6.00E-12	293	0.2	0.76	WRKY transcription factor, WRKY transcription factor
		BLINK					2.50E-15	293	0.2	1.03	
	PSTv218	FarmCPU					1.18E-08	332	0.2	0.53	
		BLINK					9.51E-13	332	0.2	0.73	
*Pgt*	QFCSC	MLM	Subpop 2	S3S_835968584	3S	835.97	4.63E-08	160	0.04	1.43	Disease resistance protein (NBS-LRR), Octicosapeptide/Phox/Bem1p (PB1) domain-containing protein/tetratricopeptide repeat (TPR)-containing protein
		FarmCPU					8.97E-26	160	0.04	1.25	
		BLINK					4.75E-15	160	0.04	1.2	
*Pt*	THBJG	FarmCPU	Whole	S3S_885155266	3S	885.16	1.38E-08	302	0.19	0.97	F-box family protein, Disease resistance protein (NBS-LRR), Pm3-like disease resistance protein (NBS-LRR)
	TBBGS	BLINK					3.90E-07	264	0.16	0.91	
	THBJG	BLINK					1.52E-10	302	0.19	1.08	
*Pgt*	TTTTF	MLM	Subpop 2	S4S_697635226	4S	697.64	1.74E-08	135	0.04	2.47	NAD(P)-linked oxidoreductase-like protein, Protein kinase, Protein FAR1-RELATED SEQUENCE 5
		FarmCPU					7.63E-18	135	0.04	1.65	
		BLINK					3.54E-32	135	0.04	3.33	
*Pgt*	TTTTF	MLM	Subpop 2	S5S_535624330	5S	535.62	1.67E-07	135	0.04	2.55	Tonoplast dicarboxylate transporter, E3 ubiquitin-protein ligase-like protein
		FarmCPU					5.86E-22	135	0.04	2.96	
		BLINK					1.44E-27	135	0.04	3.01	
*Pt*	BBBDB	MLM	Whole	SUN_49934383	UN	49.93	1.61E-08	262	0.41	2.93	F-box/FBD/LRR-like protein
		FarmCPU					2.75E-11	262	0.41	1.89	
		BLINK					4.23E-18	262	0.41	3.11	
	BBBQD	BLINK					6.59E-10	280	0.42	1.54	
*Pt*	BBBDB	BLINK	Subpop 2	SUN_232304889	UN	232.3	5.32E-07	122	0.16	1.64	F-box/FBD/LRR-like protein, Protein FAR1-RELATED SEQUENCE 6
	TBBGS	BLINK					3.20E-21	115	0.1	1.86	
	THBJG	FarmCPU					3.60E-09	138	0.13	1.14	
		BLINK					1.24E-16	138	0.13	1.83	

^a^ All GWAS models were performed independently in the whole population and in each of two subpopulations. The two subpopulations were identified via K-means clustering.

^b^ Number of accessions with observations for each trait in the relevant population(s).

^c^ MAF, minor allele frequency.

^d^ Absolute value of allelic effect size, or postulated effect of the allele on a phenotype.

e Gene annotation is according to the reference assembly of Ae. longissima (accession AEG-6782-2, Avni et al., 2022). Genes listed are within 1 Mbp of the significant marker.

One accession (AEG-2265-22) from subpopulation 1 carried eight out of the nine favorable alleles at these nine key loci. An additional ten accessions from subpopulation 1 carry seven out of the nine favorable alleles at the key loci. Subpopulation 1 had a higher frequency of resistance alleles at these loci compared to subpopulation 2, although it should be noted that no significant MTA was detected by all three GWAS models in subpopulation 1.

### 3.4 Candidate genes

One megabase sections of the genome flanking key markers were examined using the reference assembly of *Ae. longissima* (accession AEG-6782-2, Avni et al., 2022) and the UCSC Genome Browser (Kent et al., 2002). These flanking regions were investigated for annotated genes that may play a role in plant defense responses. Candidate genes near key loci identified on scaffolds unassigned to chromosome assemblies were restricted to gene annotations on the same contig as the marker. Nineteen candidate genes associated with disease resistance were identified near the nine key SNP markers (**Table 3**). Most notably, three genes annotated as nucleotide binding site leucine-rice repeat proteins (NLRs; AE.LONG.r1.3SG0251830, AE.LONG.r1.3SG0259550, AE.LONG.r1.3SG0259480) were identified near SNP markers S3S_835968584 and S3S_885155266, associated with resistance to *Pgt* race QFCSC and *Pt* races THBJG and TBBGS, respectively. Utilizing Basic Local Alignment Search Tool (BLAST), no highly similar sequences to AE.LONG.r1.3SG0259550 with high query coverage were found in related genomes. AE.LONG.r1.3SG0251830 shared 99.3% identity to a gene on chromosome 3B (TraesCS3B03G1230100.1) of wheat, while AE.LONG.r1.3SG0259480 shared 98.5% identity to a gene (AE.SHARON.r1.3SG0285570) on chromosome 3S of *Ae. sharonensis* All other candidate genes have also been reported to play a role in various defense responses (**Table 3**).

## 4 Discussion

### 4.1 Resistance to rusts of wheat in the ALDIVCO

The main objective of this study was to expand evaluations of a large and diverse panel of *Ae. longissima* accessions for resistance to stem rust, leaf rust, and stripe rust and to identify loci associated with seedling-stage resistance to these diseases. Highly variable and ever-evolving populations of the causal pathogens of these wheat rusts present a continuous challenge to breeding for durable disease resistance. The wild relatives in wheat’s secondary gene pool, such as *Ae. longissima*, can serve as valuable sources of novel resistances (Schneider et al., 2008; Olivera and Steffenson, 2009; Liu et al., 2011; Klindworth et al., 2012).

The percentage of resistant accessions in the ALDIVCO varied depending on pathogen race but was about half of the total panel (≈ 40-60%) (**Table 2**). The panel showed the widest range of frequency of resistance to the *Pgt* isolates. Frequency of resistance was lowest to *Pgt* TTTTF (18.5%), known to be the most widely virulent race of the pathogen in the United States. In contrast, frequency of resistance was nearly 100% (with only one heterogeneous accession) to *Pgt* QFCSC. Although not one accession showed susceptibility to *Pgt* QFCSC, linearization of the still variable ITs within the resistant category allowed for association mapping. The frequency of resistance was also quite high to race TTKSK (80.6%) and moderate to race TTRTF (43.7%). This large variation in the frequency of resistance across different *Pgt* races indicates strong race specificity of stem rust resistance genes in *Ae. longissima*. For leaf rust, the frequency of resistance was intermediate to all races (52.2%-68.5%). Similarly, an intermediate frequency of resistance (49.5% and 53.7%) was also observed for PSTv37 and PSTv40 of *Pst*. A higher frequency of resistance (79%) was observed for *Pst* race PSTv218. High frequencies of adult plant resistance to bulk Israeli isolates of *Pst* and *Pt* (91 and 97%) were reported by Anikster et al. (2005) in a collection of 512 *Ae. longissima* accessions. However, seedling reactions to North American isolates of *Pt* (races SBDB and TBBL) showed lower frequencies of resistance (39%) in this *Ae. longissima* panel, reflecting more closely the intermediate frequencies of *Pt* resistance against the additional North American races reported in this study. Interestingly, similar frequencies of seedling resistance to *Pgt* TTTTF (13.1%) and TTKSK (69.2%), and to *Pt* THBJG (62.6%) and BBBB (59.8%) were reported by Olivera et al. (2007) in a collection of 107 *Ae. sharonensis* accessions from Israel. *Ae. sharonensis*, resides in sympatric habitats along the Israeli coast, is a close relative of, and has been hypothesized to hybridize with *Ae. longissima* (Sela et al., 2018), which may explain the similar frequencies of resistance reported in these two species. Overall, these results demonstrate that *Ae. longissima* is a diverse and abundant source of resistance to stem, leaf, and stripe rust.

*Ae. longissima* is typically self-pollinated, although some out-crossing may occur. For these experiments, the accessions evaluated ranged from self generation S1 to S6, but after the initial generation of single seed decent, they were selfed and bulked for increase of seed stocks. Many accessions tested exhibited heterogeneous reactions (i.e. had both resistant and susceptible plants) to one or more of the pathogen races tested, suggesting that heterozygosity may be present at some resistance loci (**Table 2**). This degree of heterogeneity was notably high for certain races evaluated, including *Pt* race TBBGS (24.8%) and *Pst* race PSTv40 (31.5%), although it should be noted that these percentages included accessions that were clearly segregating for resistance (e.g. one plant was rated 1 or 0;, while another plant was rated 3 or higher) and accessions that had heterogeneous ratings that were less stark (e.g. one plant was rated 2+ while another plant was rated 3 or 3-). This variation may be due to rater inconsistency or differing environmental conditions, such as light and temperature differences, across experiments. This is especially notable for the *Pst* races evaluated, due to the increased dependence of uredinia formation on light penetration and more quantitative nature of the symptoms seen with infection by this pathogen. Similar levels of heterogeneous reactions were reported in a collection of *Ae. sharonensis* (maximum of 22.4% heterogeneous to *Pt* race BBBDB) by Olivera et al. (2007).

All ITs to pathogen races of the same species were significantly positively correlated (**Supplementary Figure S4**) in this study, which was expected due to the presence of common genetic factors controlling resistance to each pathogen. A general lack of correlation between the stem rust reactions and leaf rust reactions was observed, while a negative correlation between stem rust reactions and stripe rust reactions was found, indicating that genetic factors controlling resistance are pathogen-specific.

Geographic and genotypic patterns of resistance were observed in the ALDIVCO. Subpopulation 1, from the northern and central coastal region of Israel, exhibited a higher frequency resistance to stem and leaf rust, while subpopulation 2, mostly from southern Israel, exhibited a higher frequency of resistance to stripe rust. No significant difference was found between the subpopulations for *Pt* races TBBGS and BBBDB, but subpopulation 1 did exhibit higher frequencies of resistance to races THBJG and BBBQD. Huang et al. (2018) evaluated this geographic differentiation in populations with respect to differing levels of resistance for the three different pathogens via local indicator of spatial association (LISA) maps.

The additional races evaluated in this study showed a similar trend. As mentioned by Huang et al. (2018), the presence of higher stem rust resistance in the north-central coastal region of Israel cannot be easily attributed to coevolutionary disease selection pressure due to higher levels of precipitation or increased inoculum due to wheat cultivation. Stem rust of cultivated wheat occurs more frequently in southern Israel (H. Sela, unpublished). In contrast, a higher frequency of resistance to stripe rust was found in the southern populations, which could possibly be explained by lower minimum temperatures leading to more frequent dew periods that favor the development of stripe rust. In addition, southern desert populations of the grass mature earlier, perhaps exposing them to increased risk of infection by *Pst*. Thus, the overall different resistance spectrum of the sub-populations could be attributed to pathogen pressure.

Alternatively, the geographic patterns of rust resistance may be due to other associated adaptive traits and limited geneflow between the two subpopulations. Anikster et al. (2005) posited that long-term stand density of *Aegilops* populations might be a more important determinant of local development of rust disease than differences in climate, perhaps leading to evolution of increased resistance in sites where their populations have reached greatest density. Further investigation into the distribution of naturally occurring rust diseases on *Ae. longissima* populations in Israel may add additional context to help explain the observed pattern of resistance.

### 4.2 Population structure in the ALDIVCO

*K*-means clustering utilizing the genotypic matrix and hierarchical clustering resulted in the identification of two subpopulations, confirmed by PCA (**Figure 1**, **Supplementary Figure S3**). Population structure was associated with geographic distribution of the ALDIVCO accessions, with subpopulation 1 having been collected chiefly from the coastal area north of latitude 31.84 and subpopulation 2 mostly from the south-central coastal area south of latitude 31.84 and extending into the Negev desert. The same clear pattern of separation between northern coastal and southern desert populations of *Ae. longissima* was observed by Sela et al. (2018), in which 23 accessions were genotyped via GBS and PCA analysis was performed. In the current expanded investigation, one group of seven accessions from the eastern shore of the Sea of Galilee did not follow this latitudinal trend of relatedness; rather they were more closely related to southern accessions. The reason for their relatedness to the southern accessions is unknown but may be due to migration via animal or human movements. Some accessions were intermediate between the two subpopulations, suggesting probable admixture between the northern and southern populations. Sela et al. (2018) theorized the geographic difference in subpopulations was likely to create local adaptations because of significant differences in soil and climate. As mentioned above, phenotypic differences were observed for disease resistance to different rust pathogens between these two subpopulations.

### 4.3 Linkage disequilibrium in the ALDIVCO

The power of GWAS is dependent on LD between markers and underlying QTLs. The strength of LD between adjacent markers was relatively low (r2 = 0.17). LD decayed rapidly in a relatively short distance for all chromosomes, as expected for a diverse panel of germplasm, where accumulated historic recombination events cause a drop in LD between markers as distance increases. For chromosomes 3S, 4S, and 7S, a slight increase in LD was observed from approximately 20 Mbp to 440, 260, and 280 Mbp respectively (**Supplementary Figure S1**), followed by the expected decrease at the longest distances. It was also observed that the central regions of the chromosomes tended to have much higher LD than the distal regions (**Supplementary Figure S2B**), as expected, with lower recombination near the centromeric regions. There also tended to be much longer distances between markers near the central regions of the chromosomes (**Supplementary Figure S2A**), especially for chromosomes 3S and 4S, which could also explain the observed patterns of slight increases in LD with distance for these, as larger distances between markers in regions of high LD skews the average pairwise marker trend towards higher LD at longer distances.

### 4.4 GWAS for rust resistance in the ALDIVCO

This study is the first extensive association mapping study for wheat rust resistance in *Ae. longissima* and the first to utilize the recently completed *Ae. longissima* reference genome assembly for SNP calling and subsequent GWAS. A large number (195) of significant markers were associated with resistance to at least one of 10 of the 11 rust pathogen races evaluated with models run utilizing data from the entire population (n = 381) and each subpopulation (n = 204 and 174) independently. This large number of associations is not unprecedented; for example, Sallam et al. (2017) reported 250 significant markers associated with resistance to just five stem rust cultures in a panel (n = 314) of wild barley (*H. vulgare* subsp. *spontaneum*) and Hinterberger et al. (2022) reported 73 MTAs for resistance to one isolate of *Blumeria graminis* f. sp. *tritici* in a panel (n = 8,316) winter wheat (*T. aestivum*).

The large number of markers generated by GBS and the low LD discovered in the panel facilitated capture of many genetic variants, enabling detection of additional MTAs. However, the large proportion of missing data–which is common in many GBS marker sets–along with the non-normal distribution of the disease phenotypes may call into question the validity of some identified MTAs. For this reason, multiple models were utilized to narrow the list of candidate MTAs to those that are less likely to be stochastic or genotyping bias artifacts. The single marker MLM tended to have a worse fit and detected fewer variants than either of the two multi-locus models (only two MTAs were detected by MLM for all races in the whole population). Both multi-locus models, FarmCPU and BLINK, have improved statistical power and reduce false positives (Wang and Zhang, 2021). For simplicity in reporting and to reduce the number of candidate markers, we only investigated genomic regions flanking SNPs that were detected at least three times; by all three models, or in more than one population and by multiple models, or for more than one pathogen race and by multiple models. These key markers are more likely to be true causal variants or linked to true causal variants.

GWAS was performed in each subpopulation independently to further reduce the confounding effect of population structure and to detect variants that may be too low in allele frequency in the larger population to be detected. A MAF threshold of ≥ 0.01 was instituted in the subpopulations, which is typically lower than many standard GWAS studies because rare SNPs can cause false positives, especially for small samples. However, removal of rare SNPs also may remove true causal genetic variants. For this reason, they were retained in the analyzed dataset; however, the detected MTAs for rare alleles must be interpreted with caution. In this study, that includes the rare variants S3S_835968584, S4S_697635226, and S5S_535624330 detected in subpopulation 2 for resistance to *Pgt* races QFCSC and TTTTF (**Table 3**). For all three of these markers, accessions carrying the minor allele in subpopulation 2 did have more extreme phenotypes (higher infection types in the case of S3S_835968584 for QFCSC, and lower infection types for S4S_697635226, and S5S_535624330 for TTTTF). However, in the whole population this pattern is not observed, as individuals in subpopulation 1 carrying the relevant allele have mixed infection types. For S3S_835968584 and S5S_535624330, the minor allele frequency in the whole population is still quite low, while S4S_697635226 has a higher minor allele frequency in the whole population. There is a possibility that linkage between an underlying causative gene and the detected MTA was broken in subpopulation 1, and not in subpopulation 2. Also, there could be subpopulation-specific genetic background effects which influence the loci’s impact on the phenotype.

It is likely that all of the key MTAs reported in this study are novel associations because this is the first association mapping study of rust resistance in *Ae. longissima*. There is a possibility that some of the reported MTAs arose from underlying orthologous genes to previously reported MTAs in related species. *Sr46* from *Ae. tauschii*, and *Sr39* and *Sr47* from *Ae. speltoides* have been mapped to chromosomes 2D and 2S (Faris et al., 2008; Yu et al., 2010; Arora et al., 2019), while in this study two key MTAs for resistance to *Pt* and *Pgt* were found on chromosome 2S. Both *Sr46* and *Sr39* were located on the short arms of the chromosome, while the two associations reported here reside near the centromeric region and on the long arm of chromosome 2S. However, *Sr47* was also found on the long arm of chromosome 2S. Similarly, leaf rust and stripe rust resistance loci have been reported on the short arms of chromosomes 3D and 3S in *Ae*. *tauschii* and *Ae. searsii* (Liu et al., 2011; Sharma et al., 2022), while the three MTAs reported in this study on chromosome 3S (two for resistance to stem rust and one to leaf rust) reside on the long arm. The molecular relationship, if any, between the previously reported rust resistance genes in section Sitopsis species, wheat, or other relatives and the MTAs reported here in *Ae. longissima* will require fine mapping and further comparative studies.

Four of the nine key MTA reported in this study were detected for resistance to more than one rust race within a pathogen species (specifically *Pst* and *Pt*). The underlying causative genes associated with these MTAs may be more broadly effective against more pathogen races, and thus may be more valuable for future study and breeding efforts.

### 4.5 Candidate genes

A 2-Mbp region surrounding each of the nine key SNPs identified was investigated for the presence of annotated genes that may be involved in a disease resistance response (**Table 3**). The marker S2S_363051049 associated with *Pt* TBBGS resistance on chromosome 2S resides near the putative gene AE.LONG.r1.2SG0110730, annotated as a cyclic nucleotide-gated cation channel alpha-3. These types of proteins are involved in the activation of defense responses and hypothesized to be pathogen-inducible Ca^2+^ channels that help lead to the induction of the oxidative burst and protein kinase cascades that occur upon recognition of pathogen elicitors (Moeder et al., 2011). Also on chromosome 2S, marker S2S_602532465, associated with resistance to *Pgt* TTKSK, was near two genes (AE.LONG.r1.2SG0127680, AE.LONG.r1.2SG0127730) which are F-box domain (FBD) family proteins, and near a putative LURP-one-like protein (AE.LONG.r1.2SG0127780). FBD proteins are involved in selective protein ubiquitination and subsequent degradation, a key regulatory mechanism in plants. Certain FBD proteins have been shown to contribute to resistance against pathogen infections, providing tolerance to apoplastic reactive oxygen species (ROS), regulating defense signaling, and repressing pathogen signaling (Piisilä et al., 2015; Hedtmann et al., 2017; Zhang et al., 2019). The LURP-one related (LOR) proteins LURP1 and LOR1 in Arabidopsis were shown to be required for basal defense against *Hyaloperonospora arabidopsis* (Baig, 2018). Marker S3S_669959829 on chromosome 3S, associated with resistance to *Pst* PSTv40 and PSTv218, is 98 kb from AE.LONG.r1.3SG0233030 and 428 kb from AE.LONG.r1.3SG0233070, genes both annotated as WRKY transcription factors. These transcription factors play a well-documented role in the downstream signaling post direct or indirect interactions with pathogen associated molecular patterns (PAMPS) or effector proteins (Wani et al., 2021). In wheat, two WRKY genes were identified in association with high-temperature seedling plant (HTSP) resistance to *Pst* by RNA-seq and were found to both negatively and positively regulate HTSP resistance via differential regulation of salycilic acid- (SA), jasmonic acid- (JA), ethylene-, and ROS-mediated signaling (Wang et al., 2017). Additionally, two different WRKY transcription factors in wheat were found to interact with a LRR receptor-like kinase (RLK), to function as positive regulators of HTSP resistance to *Pst* (Wang et al., 2019).

Many race-specific plant disease resistance genes belong to the nucleotide-binding site leucine-rich repeat (NLR) gene family, encoding proteins that recognize pathogen effectors and trigger ETI, which often includes a hypersensitive response of localized cell death, as observed in resistant wheat rust infection types (Sánchez-Martín and Keller, 2021). Marker S3S_ 835968584 on chromosome 3S, associated with resistance to *Pgt* QFCSC is near a putative NLR (AE.LONG.r1.3SG0251830). This locus may be orthologous to a similar (99.3% identity) gene on chromosome 3B of wheat, also a putative RGA2-like disease resistance protein. Interestingly, this locus was identified as significant only in subpopulation 2 and is responsible for solely quantitative differences of resistance to *Pgt* QFCSC, as no accessions were fully susceptible (i.e., IT ≥ 3-) to this race. There are cases in which typical NLRs or alleles of NLRs involved in qualitative resistance have been reported to confer a less strong but still effective response during quantitative resistance (Debieu et al., 2016; Díaz-Tatis et al., 2022). Another possible candidate gene near marker S3S_ 835968584 which may contribute to quantitative differences in resistance phenotypes is an Octicosapeptide/Phox/Bem1p (PB1) domain-containing protein/tetratricopeptide repeat (TPR)-containing protein. Proteins carrying the TPR domains have been shown to contribute to chitin-induced immunity and mRNA turnover of defense-related genes (Zhou et al., 2018). On chromosome 3S, marker S3S_885155266 was found significantly associated with two races of *Pt*, THBJG and TBBGS, and is near two genes annotated as NLR proteins (AE.LONG.r1.3SG0259550 and AE.LONG.r1.3SG0259480). The first gene is likely unique to *Ae. longissima*, as upon searching related genomes via BLAST, no similar sequences were found. The other gene may be orthologous to a similar (98.5% identity) gene on chromosome 3S of *Ae. sharonensis*. Additionally, this marker associated with *Pt* resistance resides near an F-box protein, another potential candidate gene related to disease resistance responses. Two markers were found associated with resistance to *Pgt* TTTTF, one on chromosome 4S (S4S_697635226) and one on chromosome 5S (S5S_535624330). The 4S marker resides near a putative NAD(P)-linked oxidoreductase-like protein (AE.LONG.r1.4SG0320810), a type which may be involved in controlling ROS accumulation during stress responses, including pathogen infection (Kant et al., 2019). Another candidate gene near this marker is annotated as the protein FAR1-RELATED SEQUENCE 5 (AE.LONG.r1.4SG0320750). FAR1 family proteins are transcription factors that have been shown to negatively regulate ROS accumulation and oxidative stress-induced cell death. Moreover, loss of function of FAR1 was shown to enhance the expression of defense-responsive genes in Arabidopsis (Ma and Li, 2018). The 5S marker associated with resistance to *Pgt* TTTTF resides in the coding region for a gene (AE.LONG.r1.5SG0395590) annotated as a tonoplast dicarboxylate transporter. This protein is a vacuolar malate transporter, which controls cytoplasmic concentration of organic acids required for many metabolic processes. There is a dearth of information in the literature regarding the role of this type of protein in plant defense, although it is known that the hormone γ-aminobutyric acid (GABA) involved in stress responses, regulates malate flux from wheat roots under acidosis and other stresses (Ramesh et al., 2015). Two markers were detected on contigs unassigned to any chromosome scaffold (chromosome UN), both associated with resistance to *Pt*, one to races BBBDB and BBBQD (SUN_49934383) and the other to races BBBDB, TBBGS, and THBJG (SUN_232304889). Both of these markers were on contigs containing genes annotated as F-box/FBD/LRR-like proteins (AE.LONG.r1.UnG0607500 and AE.LONG.r1.UnG0627200). As mentioned above, FBD proteins play key roles in regulation of defense mechanisms and signaling. One maps to a highly similar sequence on chromosome 6D of *Ae. tauschii* and the second maps to chromosome 2B on the *T. dicoccoides* genome. Additionally, marker SUN_232304889 is on the same contig as the annotated protein FAR1-RLATED SEQUENCE 6, a putative gene sharing 99.4% identity to a gene on chromosome 4S of *Ae. sharonensis*.

Collectively, all the aforementioned genes are candidates for further study relating to wheat rust resistance, however the strength of any one candidate cannot be determined from this study. Without higher resolution mapping and validation, the roles of reported genes near significant markers remain highly speculative.

### 4.6 Implications for breeding

Identification of novel sources of resistance and subsequent introgression of new resistance alleles into wheat cultivars is necessary for maintaining the sustainability of rust resistance. As rust pathogens overcome many of the commonly deployed resistance genes, the reservoir of readily accessible resistance genes from the primary genepool of wheat has become depleted, highlighting the importance of members of the secondary gene pool as sources for novel resistances.

Transfer of resistance genes from *Ae. longissima* into hexaploid wheat is a difficult process, requiring time-consuming wide-hybridization techniques. Ceoloni et al. (1992) induced recombination between wheat and *Ae. longissima*, allowing for transfer of chromosome segments from *Ae. longissima* carrying the powdery mildew resistance gene *Pm13* which has recently been cloned and shown to be a unique mixed lineage kinase domain-like (MLKL) protein (Liu et al., 2023 preprint). Leaf rust and stripe rust resistance genes *Lr56* and *Yr38* were introgressed into wheat from *Ae. sharonensis*, a closely related species to *Ae. longissima* (Marais et al., 2006). Additional leaf and stripe rust resistance genes were transferred from *Ae. sharonensis* into wheat by Millet et al. (2014). *Ae. longissima* accessions showing resistant reactions to all of the pathogens evaluated in this study and/or carrying multiple resistance alleles from identified MTAs would be ideal candidates for additional introgression studies with wheat. Likewise, the identified resistance loci and marker alleles could serve to assist in the introgression process with targeted selection and confirmation of the resistance.

Direct cloning of resistance genes from secondary genepool members and their transfer into wheat is a promising alternative to wide-hybridization. Novel methods of resistance gene cloning have sped up the process of engineering resistance in domesticated crops. For example, Arora et al. (2019) cloned four *Sr* genes from the wheat D genome progenitor, *Ae. tauschii*, utilizing association genetics with R gene enrichment sequencing (AgRenSeq). Yu et al. (2022) reported a similar success in a method not limited to identifying NLR-type genes which utilized positional mapping, mutagenesis, and RNA-Seq (MutRNA-Seq) to clone the stem rust resistance gene *Sr62* from *Ae. sharonensis*. This gene was transformed into wheat and functionally validated for its resistance to 11 *Pgt* races. Whole-genome sequencing combined with association mapping was also successfully applied for rapid cloning of *SrTA1662* in *Ae. tauschii* (Gaurav et al., 2021). The marker-trait associations reported in this study may serve as promising leads for additional gene cloning efforts in *Ae. longissima*, with the markers residing near putative NLR and FBD-LRR proteins as key candidates for investigation.

Whole genome sequencing of a subset of the ALDIVCO is underway to obtain a more robust genotypic dataset, which will allow for more precise identification of candidate genes associated with rust resistance. Additionally, a smaller subset of accessions will be sequenced to greater depth for the creation of an *Ae. longissima* pangenome to facilitate investigation of presence/absence and other structural variation in the genomes of this species.

Through this study we find that *Ae. longissima* is a rich source of diverse resistance to the devastating rust pathogens of wheat. The developed genomic resources along with genetic information such as the disease resistance MTAs reported here can lead to accelerated development of improved wheat germplasm with novel resistance from these secondary gene pools. This will be critical to the continual efforts to protect the world wheat supply from rapidly evolving pathogens.

## Data availability statement

The raw data supporting the conclusions of this article will be made available by the authors, without undue reservation.

## Author contributions

RP carried out phenotyping, analyzed the phenotype and genotype data, performed genome-wide association mapping, and wrote the draft of the manuscript; SH carried out phenotyping and assisted in assembly of the germplasm panel; SS helped in the SNP detection from the GBS data; AS supervised the selfing and production of germplasm stock; MR and HS produced and maintained the germplasm stocks; HS assisted with GWAS validations; JP supervised the genotyping by sequencing of the panel; BS supervised the study and secured funding. All the authors have read the manuscript and approve it.
