# Supplementary material for: Genome-wide association mapping of rust resistance in Aegilops longissima
Source: Front Plant Sci. 2023 Jul 27;14:1196486. doi: 10.3389/fpls.2023.1196486 (PMC10413114; doi:10.3389/fpls.2023.1196486)
Supplement: Supplementary file 1 [file DataSheet_1.pdf]

Supplementary Material

Genome-wide association mapping of rust resistance in *Aegilops longissima*

Rae Page, Shuyi Huang, Moshe Ronen, Hanan Sela, Amir Sharon, Sandesh Shrestha, Jesse Poland, and Brian J. Steffenson*

*** Correspondence:** Corresponding Author: bseffen@umn.edu

1 SUPPLEMENTARY FIGURES

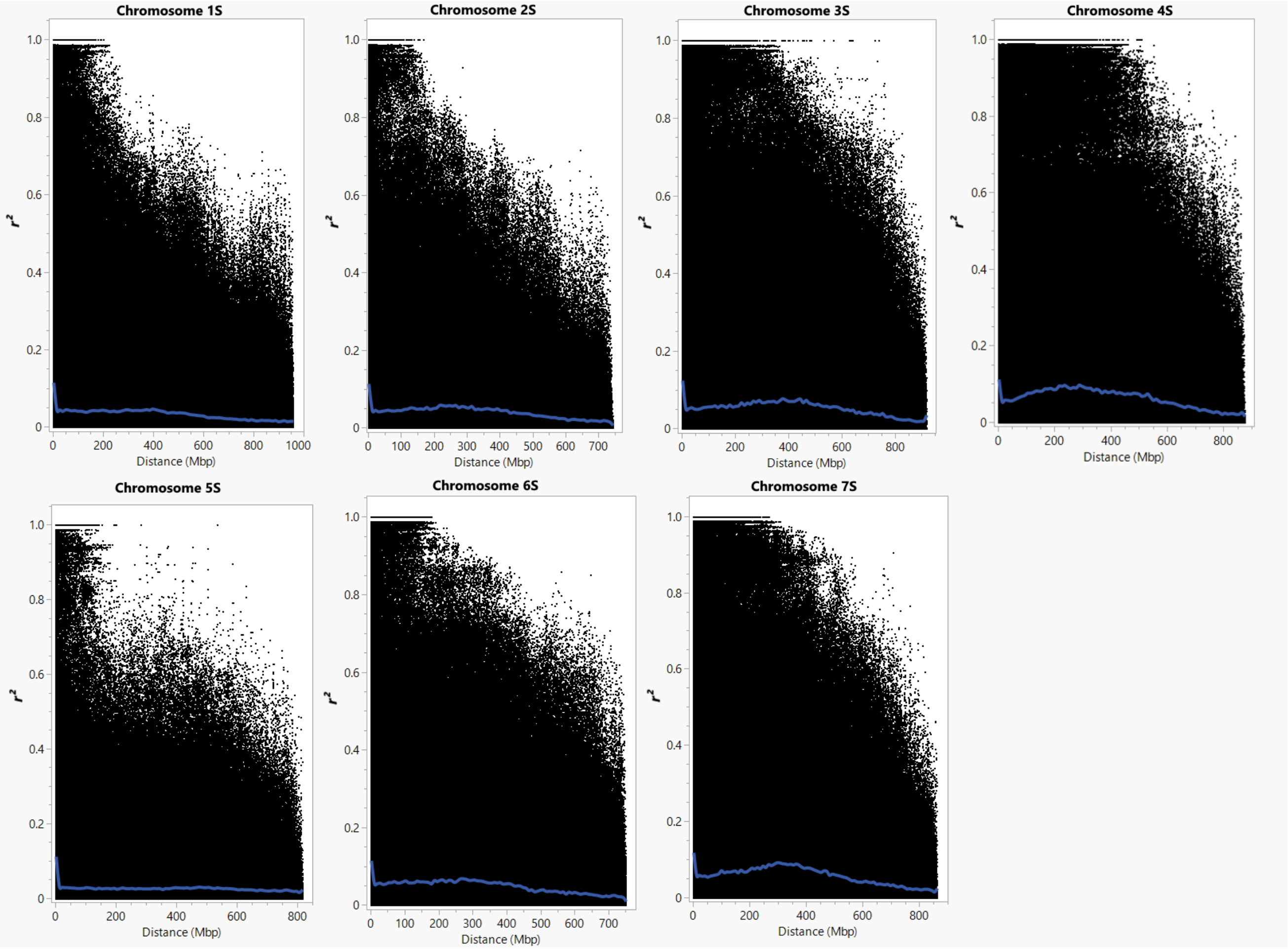

Supplementary Figure S1. Linkage disequilibrium decay for each chromsome for 381 *Ae. longissima* accessions of the ALDIVCO estimated as r^2 using full matrix of marker comparisons for markers with < 20% missing data.

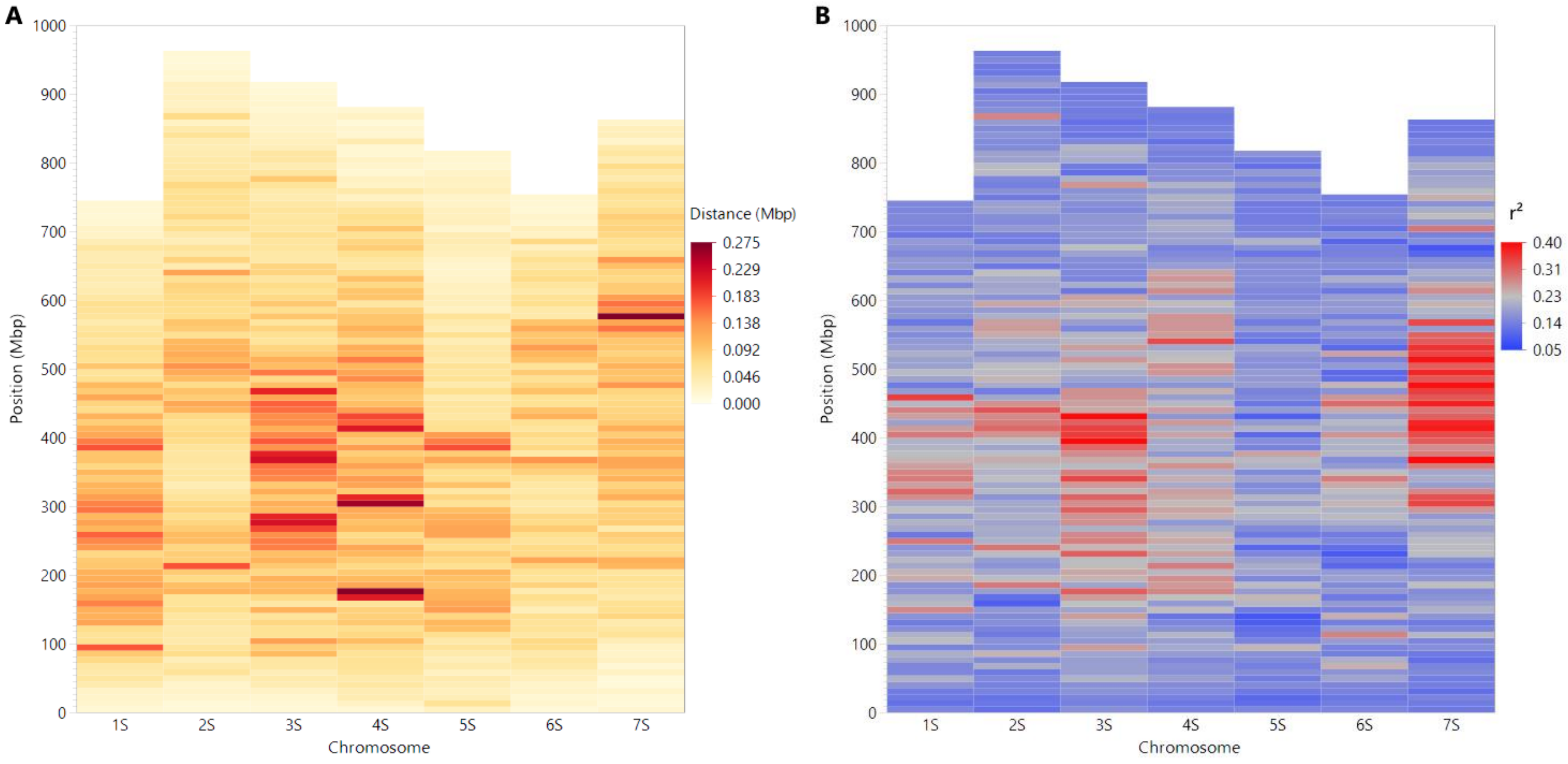

Supplementary Figure S2. (A) Average distance between adjacent markers and **(B)** average adjacent marker linkage disequilibrium (LD) across all seven chromosomes.

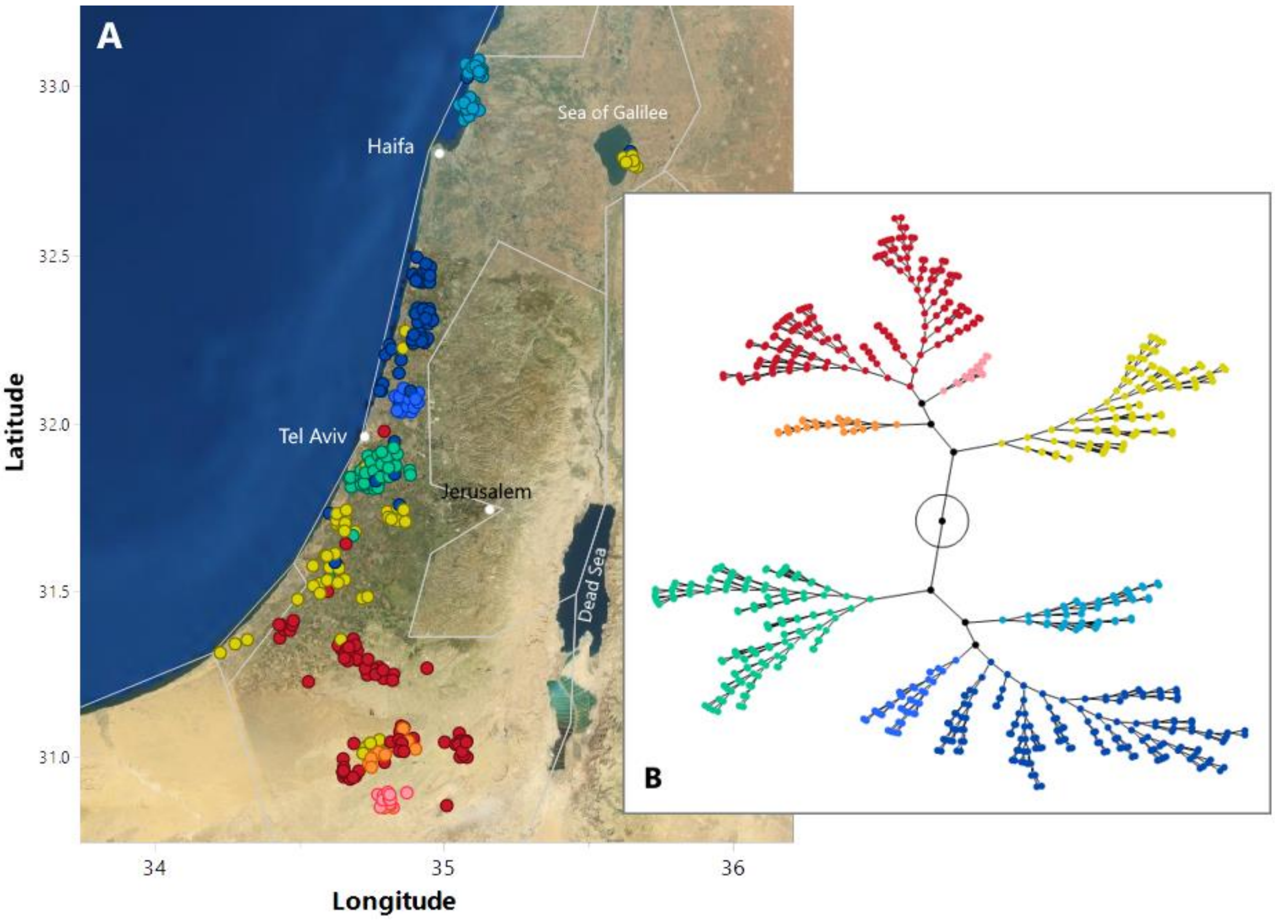

Supplementary Figure S3. (A) Geographic distribution of accessions in the ALDIVCO colored according to subpopulations identified via hierarchical clustering using Ward's minimum variance method on the genetic distance matrix and **(B)** constellation plot indicating genetic relatedness among accessions.

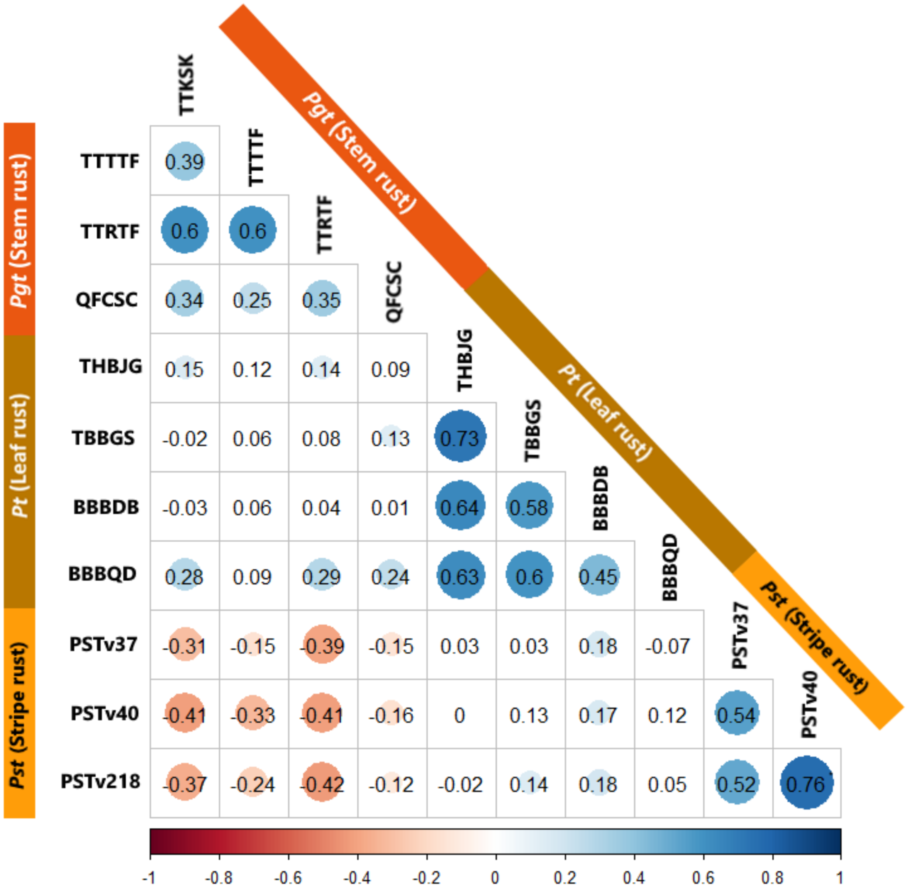

Supplementary Figure S4. Correlation coefficients for mean linearized infection types between 11 different wheat rust pathogen races in the ALDIVCO. The darker blue color indicates a higher positive correlation, while a darker red color indicates a more negative correlation. Correlations with no colored circle present are not significant ($p > 0.05$).

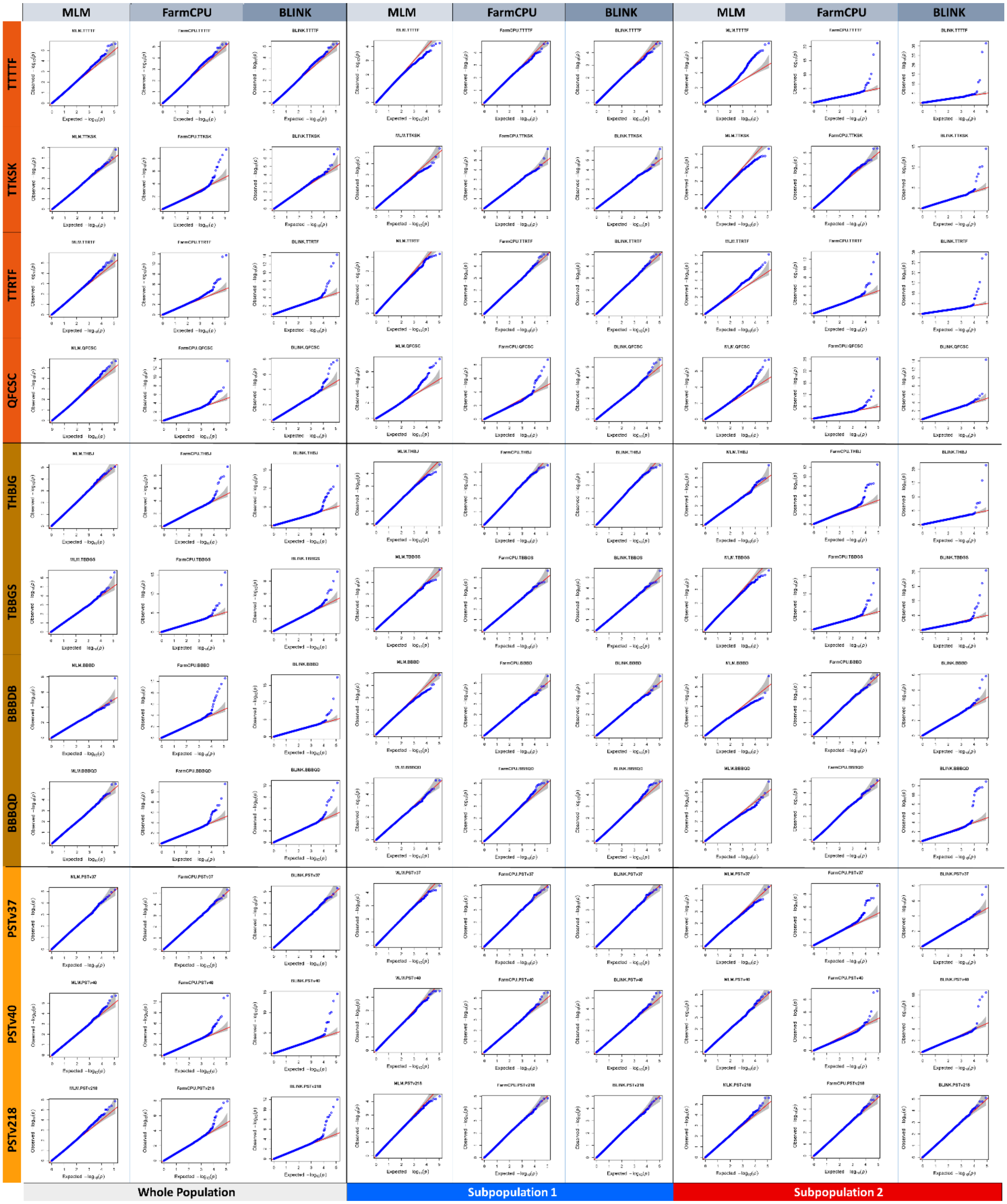

Supplementary Figure S5. Quantile-quantile (QQ) plots for observed *p*-values of three GWAS models (MLM, FarmCPU, and BLINK) utilized to identify associations in the ALDIVCO for seedling resistance against four races of the stem rust pathogen (*Puccinia graminis* f. sp. *tritici*), four

races of the leaf rust pathogen (*Puccinia triticina*); and three races of the stripe rust pathogen (*Puccinia striiformis* f. sp. *tritici*). Models were run separately for a) the whole population of accessions, b) subpopulation 1 and c) subpopulation 2. The solid red line shows the expected null distribution of *p*-values assuming no associations.
